# Effects of strength training and raloxifene on femoral neck metabolism and microarchitecture of aging female Wistar rats

**DOI:** 10.1038/s41598-017-13098-5

**Published:** 2017-10-31

**Authors:** Camila Tami Stringhetta-Garcia, Samuel Rodrigues Lourenço Morais, Fernanda Fernandes, Melise Jacon Perez-Ueno, Ricardo de Paula Almeida, Mário Jefferson Quirino Louzada, Antonio Hernandes Chaves-Neto, Edilson Ervolino, Rita Cássia Menegati Dornelles

**Affiliations:** 1 Programa de Pós-Graduação Multicêntrico em Ciências Fisiológicas, Departamento de Ciências Básicas, Araçatuba, 16018-805 Brazil; 2 Univ Estadual Paulista (Unesp), Faculdade de Odontologia, Departamento de Ciências Básicas, Araçatuba, 16018-805 Brazil

## Abstract

The aim of this study was to prevent female osteoporosis using strength training (ST), raloxifene (Ral) or a combination of ST plus Ral during the natural female aging process, specifically in the periestropause period. For a total of 120 days, aging female Wistar rats at 18-21 months of age performed ST on a ladder three times per week, and Ral was administered daily by gavage (1 mg/kg/day). Bone microarchitecture, areal bone mineral density, bone strength of the femoral neck, immunohistochemistry, osteoclast and osteoblast surface were assessed. We found that the treatments modulate the bone remodeling cycle in different ways. Both ST and Ral treatment resulted in improved bone microarchitecture in the femoral neck of rats in late periestropause. However, only ST improved cortical microarchitecture and bone strength in the femoral neck. Thus, we suggest that performing ST during the late period of periestropause is a valid intervention to prevent age-associated osteoporosis in females.

## Introduction

Annually, there are more than 8.9 million bone fractures due to osteoporosis worldwide^1,2^. A 240% increase in the incidence of worldwide hip fractures is estimated by 2050^3^, along with a 400–700%^4^ increase of fractures in Latin America because of population aging. Osteoporotic fractures result in lengthy hospital stays^5^, and among these fractures, a femoral neck injury is present in 75% of affected women^4^ and is associated with mortality along with varying degrees of morbidities^6^.

Estrogen deficiency that affects women during menopause is directly related to low bone mass, deterioration of bone microarchitecture^7^, associated with increased resorption and changes in the physiological bone remodeling cycle^8^. Steroid stimulating proteins such as receptor activator of NF kappaβ ligand (RANKL) and Osteoprotegerin (OPG)^9^ are crucial to the regulation of osteoclast differentiation, whereas transcription factors such as runt-related transcription factor 2 (Runx2)^10^ are important for osteoblasts. Bone matrix proteins such as alkaline phosphatase (ALP), bone sialoprotein (BSP) and osteocalcin (OCN) regulate bone mineralization. With the decrease in estrogen concentration, there is an imbalance in this bone signaling cascade: osteoclast survival increases, while osteoblast number decreases, and that results in more resorbed bone and less newly formed bone with a consequent negative balance in multinucleated basic units (BMUs)^11,12^. In BMUs, osteoblasts and osteoclasts belong to a single temporary structure and the synchronous activities of these cells are essential for maintaining bone homeostasis^12^.

Among the options for the prevention and control of osteoporosis are physical exercise^13,14^ and medication^14–17^. Raloxifene hydrochloride (Ral) is a selective estrogen receptor modulator (SERM) that displays agonist activity of the estrogen receptor (ER) in the bone tissue^18^ and prevents bone loss^14^. Regarding exercises, strength training (ST) is a simple, easily accessible, and cost-effective way to prevent the development of osteoporosis due to hypoestrogenism^14^. Data from our lab showed that ST was able to prevent bone loss in aging ovariectomized rats, evidenced by microstructural, densitometry and biomechanical improvement^14^, even though there is a decreased ER presence in aged rat bone cells, which could have decreased the cellular response to mechanical load and not have avoided the bone deterioration^19–21^.

Despite evidence of the negative effects of aging in the bones, there are few studies on primary osteoporosis that use naturally aged rats, which have a higher bone mass acquired^22^. Therefore, mechanisms of aging are still not entirely clear. Thus, we hypothesized that the damage to bone tissue, frequent in the natural female aging process, can be partially reversed with strength training, and that this reversal can be potentiated with the use of Ral plus ST. In the present study, we investigated the action of ST and Ral on bone microarchitecture, density, strength and proteins of aged rats.

## Results

### Physiological Parameters

The estrous cycle analysis of 17-month-old Wistar rats showed that the initial change that characterizes the period of periestropause in these animals was marked by increased variability in the length of the estrous cycle phases, with persistent diestrus lasting 10–12 days longer, with recurrence within 3 or 4 cycles. After a 120-day intervention, no significant main effects caused by ST or Ral and no significant interactions (ST*Ral) in body weight, uterus weight and estradiol plasma concentration were observed in 21-month-old rats (*p* > 0.05, Table 1).

**Table 1:** Final body weight, uterine weight/100 g of body weight and estradiol plasma concentration (n = 10) of aged female Wistar rats (21 month) that performed or not strength training, treated for 16 weeks with Veh or Ral (n = 10).

Group	Final Weight (g)	Uterus Weight (g)	Estradiol (pg/mL)
NT-Veh	377.2 ± 19.93	0.1817 ± 0.005	32.28 ± 1.35
NT-Ral	376.0 ± 11.66	0.1910 ± 0.009	31.87 ± 2.29
ST-Veh	371.3 ± 8.75	0.1550 ± 0.016	32.91 ± 1.15
ST-Ral	323.3 ± 14.59	0.1450 ± 0.009	33.25 ± 0.45

Statistical analysis was performed with two-way ANOVA, followed by Tukey post-hoc testing (p < 0.05) to analyze the effect of strength training (ST) and raloxifene (Ral) treatment, and any interactions (ST*Ral). Abbreviations: Veh = vehicle, Ral = raloxifene, NT-Veh = non-trained and treated with vehicle; NT-Ral = non-trained and treated with raloxifene; ST-Veh = strength training and treated with vehicle; ST-Ral = strength training and treated with raloxifene.

### Measurement of plasma levels of tartrate-resistant acid phosphatase (TRAP) and alkaline phosphate activity (ALP)

**Figure 1:**
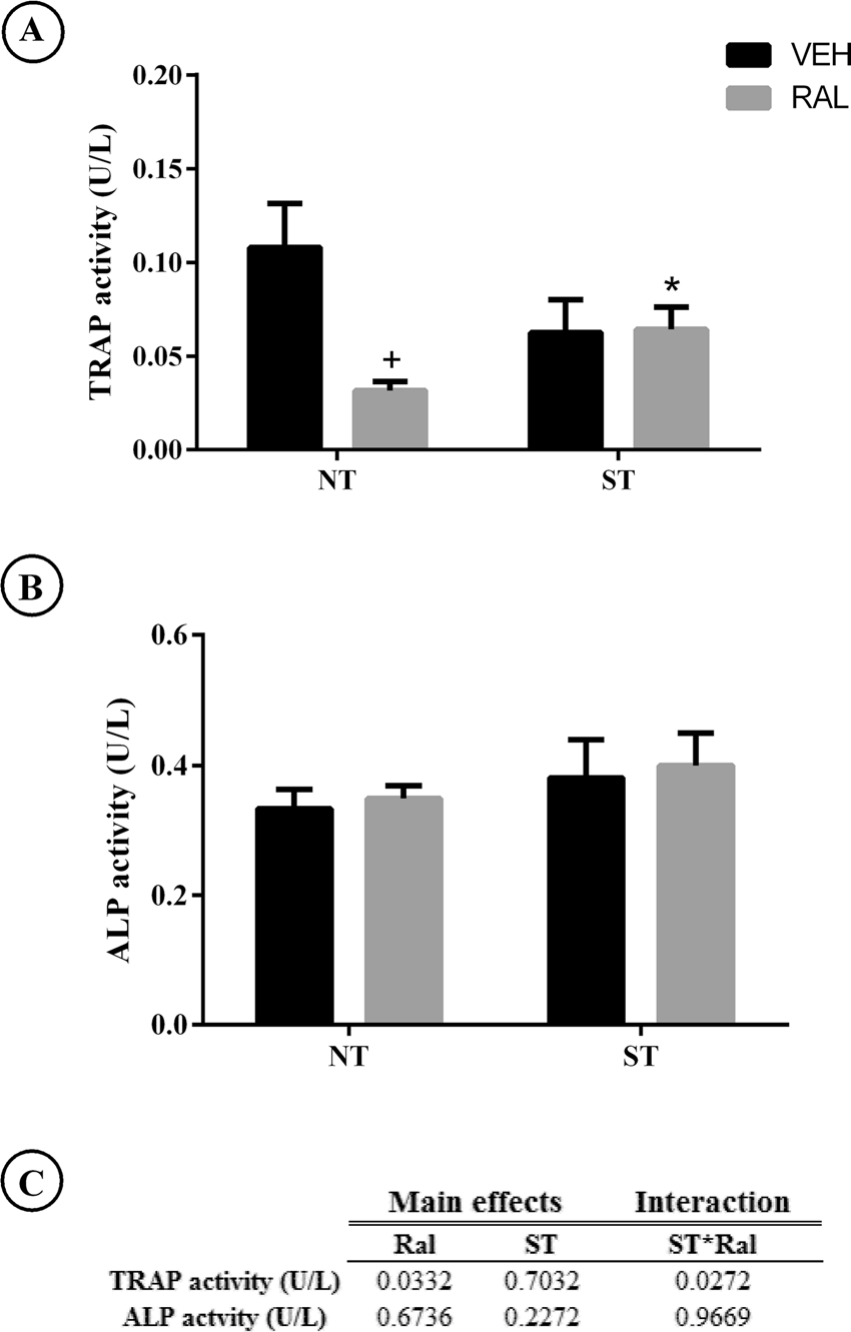
Plasma bone biomarkers in aged female Wistar rats after ST realization, Ral treatment or association of ST and Ral. (**A**) TRAP, (**B**) ALP and (**C**) summary of p values in two way ANOVA analysis, n = 8–10 animal/group. Abbreviations and symbols: +main effect of Ral, *interaction of ST plus Ral. TRAP = tartrate-resistant acid phosphatase and, ALP = alkaline phosphates, ST = strength training, Ral = raloxifene, NT-Veh = non-trained and treated with vehicle; NT-Ral = non-trained and treated with raloxifene; ST-Veh = strength training and treated with vehicle; ST-Ral = strength training and treated with raloxifene.

A summary of the plasma bone biomarker levels in each treatment group is shown in Fig. 1. Main effects of Ral treatment (NT-Ral, p = 0.0332) and interaction ST*Ral (F_(1,21)_ = 5.638, p = 0.0272) was observed in TRAP activity. ALP activity was not affected by treatments.

### Femoral neck microarchitecture after ST or Ral treatment

**Figure 2:**
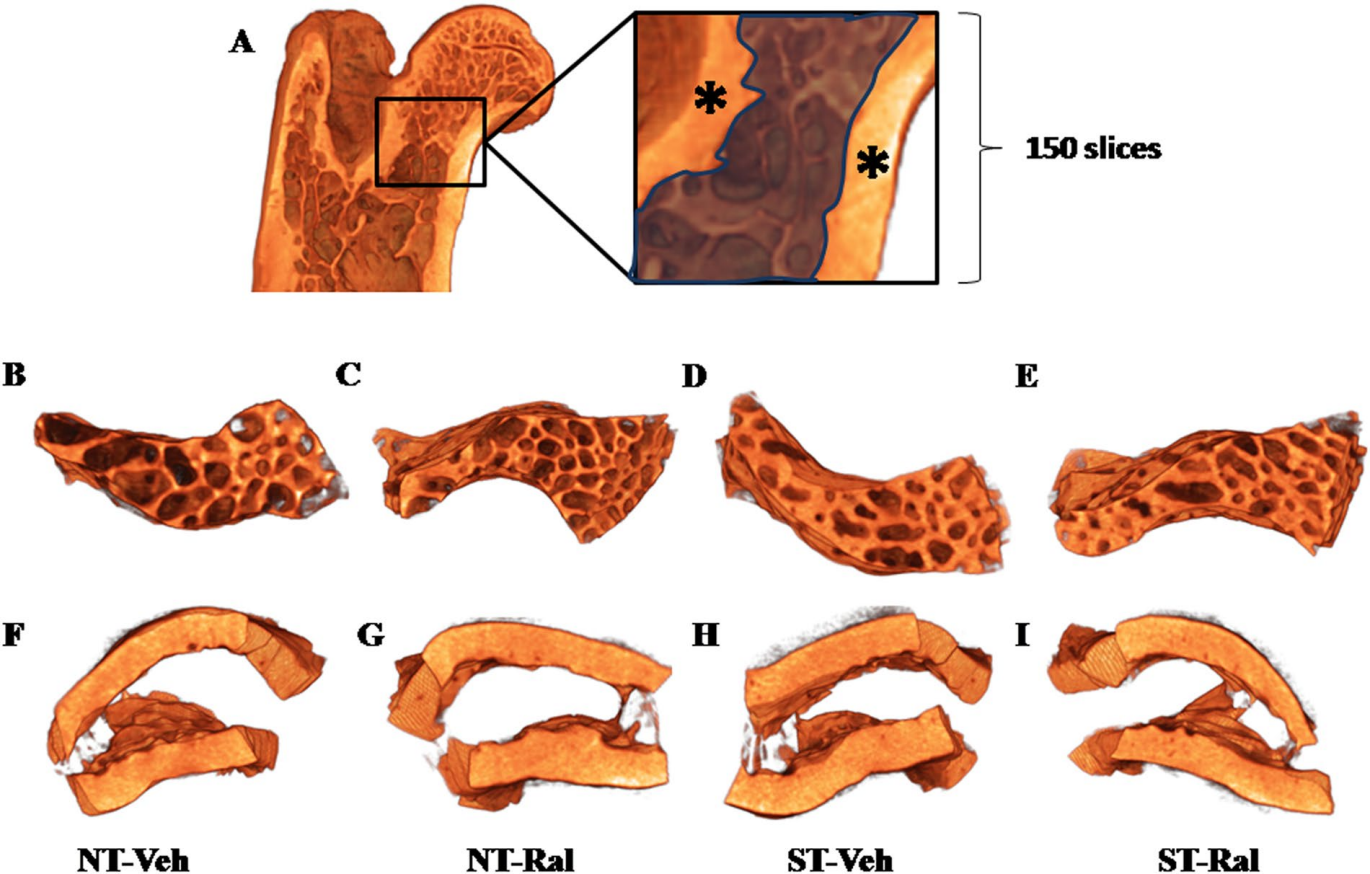
Characterization of 3D morphometric analysis. (**A**) Scanned image of proximal epiphysis, with dimensions of the region of interest (femoral neck) indicated. The region of interest in the trabecular femoral neck is marked by crosshatched and the cortical femoral neck is marked by*. The 3D images show a typical example of trabecular and cortical bone in the femoral neck of animals that did not undergo strength training (NT-Veh – **B** and **F**), animals who did not undergo strength training but received raloxifene (NT-Ral – **C** and **G**), animals who underwent strength training (ST-Veh – **D** and **H**) and animals who underwent strength training and received raloxifene (ST-Ral – **E** and **I**).

To determine whether ST, Ral or ST*Ral could influence the bone microarchitecture of femoral neck we performed micro-CT and evaluated standard structural parameters of trabecular bone as well as parameters of cortical bone in this region. The representative 3D reconstructed micro-CT images and the impact of ST, Ral treatment or ST*Ral for 120 days in femoral neck microarchitecture of aging female Wistar are shown in Fig. 2.

**Figure 3:**
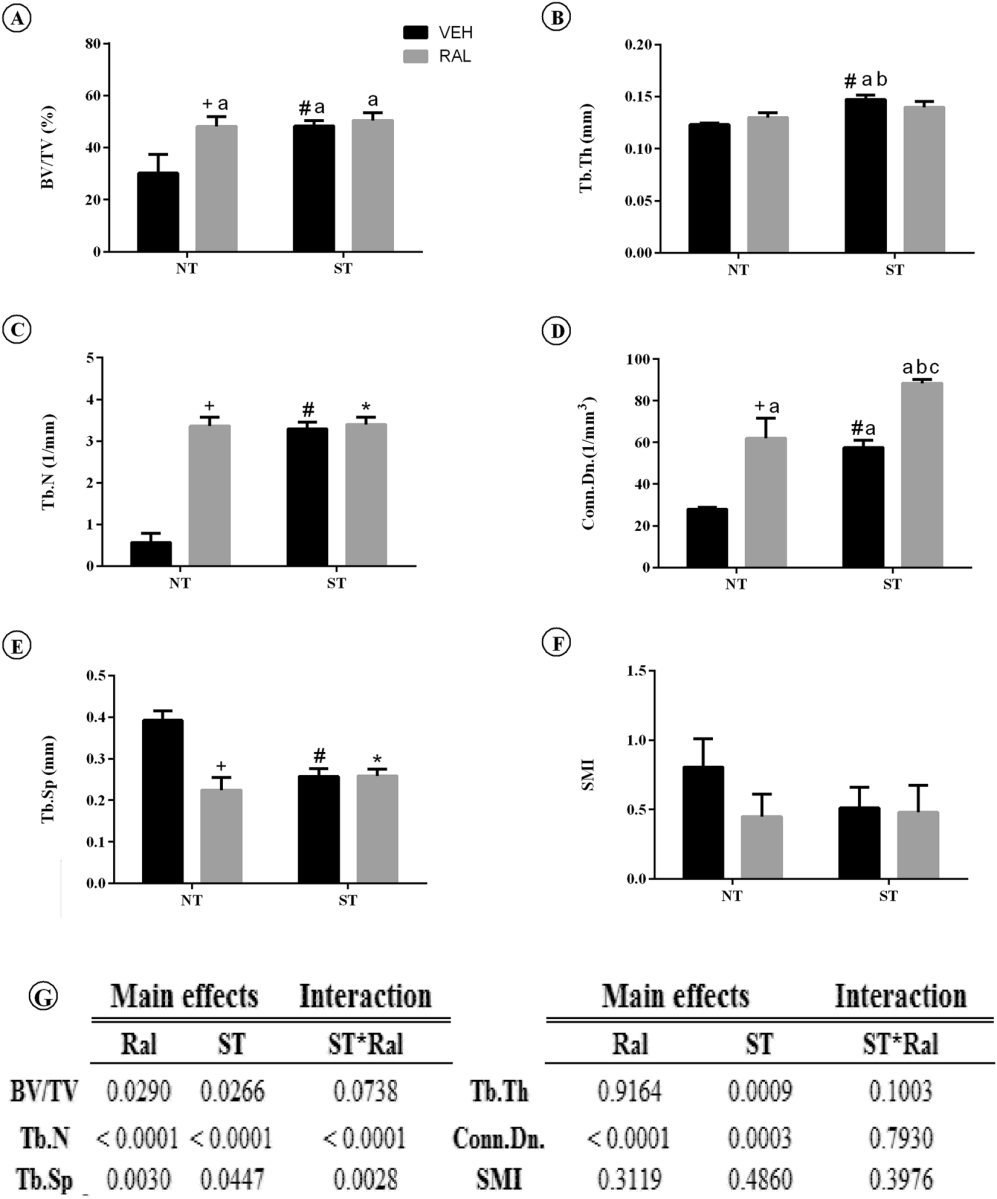
*Ex vivo* trabecular bone microarchitecture. (**A**) BV/TV, (**B**) Tb.Th, **(C**) Tb.N, (**D**) Conn.Dn, (**E**) Tb.Sp, (**F**) SMI, (**G**) summary of p values in two way ANOVA analysis, n = 7 animals/group. Data of the femoral neck assessed by microCT. Each column represents mean ± standard error of the mean (SEM). Statistical analysis was performed with two-way ANOVA, followed by Tukey post-hoc testing (p < 0.05) to analyze the effect of strength training (ST) and raloxifene (Ral) treatment, and any interactions (ST*Ral). Abbreviations and symbols: +main effect of Ral, #main effect of ST, *interaction of ST plus Ral^a^, vs NT-Veh; ^b^vs NT-Ral; ^c^vs ST-Veh. NT-Veh = non-trained and treated with vehicle; NT-Ral = non-trained and treated with raloxifene; ST-Veh = strength training and treated with vehicle; ST-Ral = strength training and treated with raloxifene; BV/TV = bone volume fraction; Tb.Th = trabecular thickness; Tb.N = trabecular number; Conn.Dn = connectivity density; Tb.Sp = trabecular separation; SMI = structure model index.

The analysis of bone volume fraction (BV/TV) in trabecular femoral neck (Fig. 3) showed significant effects after Ral treatment (p = 0.0290) and ST (p = 0.0266), but no interaction was observed. The *post hoc* analysis showed that NT-Ral (p = 0.0359), ST-Veh (p = 0.0338) and ST-Ral groups (p = 0.0243) have higher BV/TV compared to NT-Veh group. ST had main effects on trabecular thickness (Tb.Th), but Ral did not, and no interaction between treatment effects was observed. Therefore, ST-Veh group had higher Tb.Th than NT-Veh (p = 0.0041) and NT-Ral groups (p = 0.0416). In this study, it was verified an interaction of Ral and ST on trabecular number (Tb.N; F_(1,15)_ = 42.92, p < 0.0001) and separation (Tb.Sp; F_(1,12)_ = 14.06, p = 0.0028) as well as effects of Ral (p < 0.0001 and p = 0.0030) and ST (p < 0.0001 and p = 0.0447) in two way ANOVA analysis. However, no interactions of Ral and ST in Conn.Dn were revealed, although the effect of Ral (p < 0.0001) and ST (p = 0.0003) are significant. The *pos hoc* test showed that Conn.Dn was higher in NT-Ral (p = 0.0039), ST-Veh (p = 0.0238) and ST-Ral groups (p < 0.0001) compared to the NT-Veh group. SMI was not affected by treatments.

**Figure 4:**
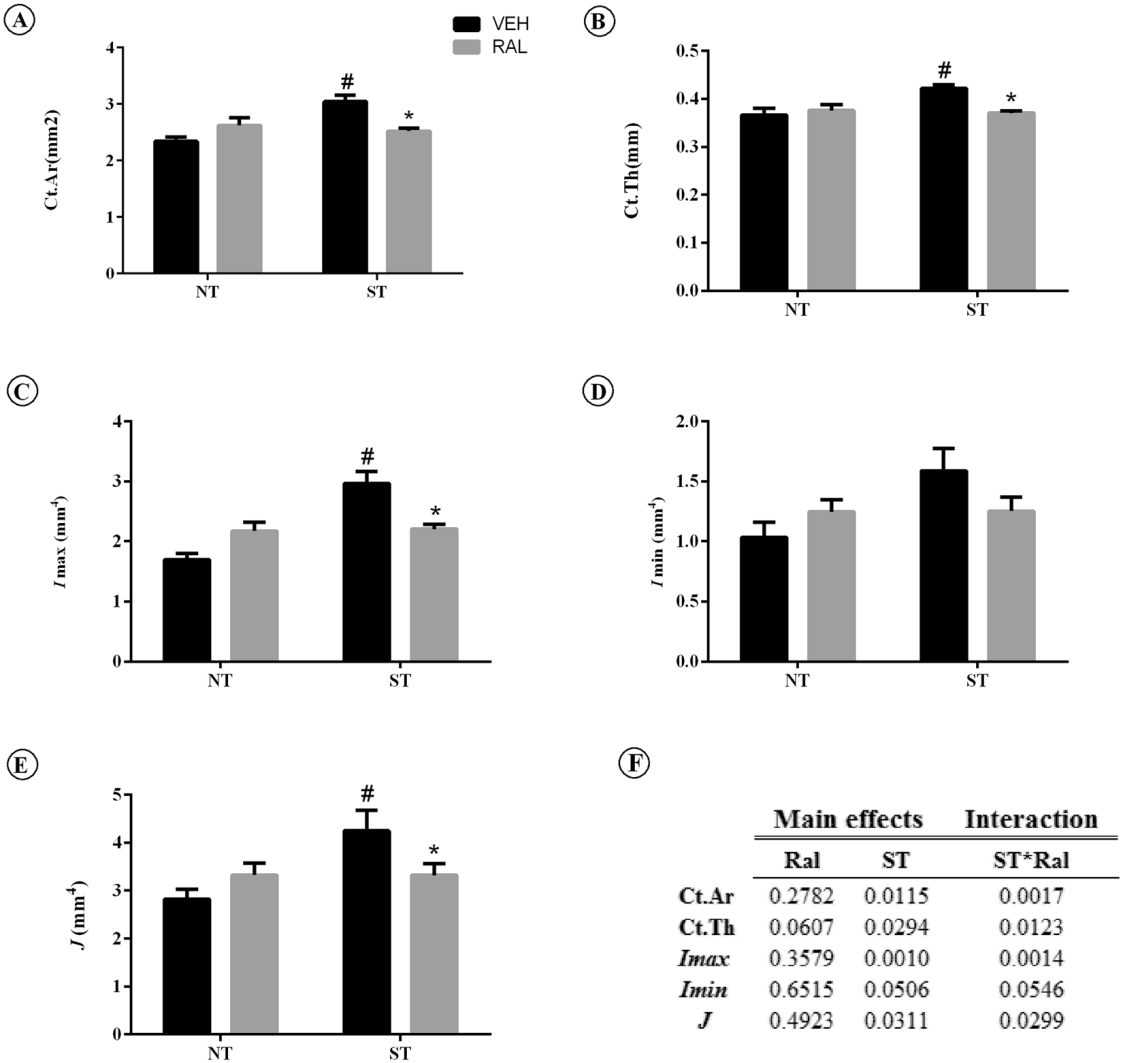
*Ex vivo* cortical bone microarchitecture. Data of the femoral neck assessed by microCT. (**A**) Ct.Ar, (**B**) Ct.Th, **(C**) *I*max, **(D**) *I*min, (**E**) J, **(F**) summary of p values in two way ANOVA analysis, n = 7 animals/group. Each column represents mean ± standard error of the mean (SEM). Statistical analysis was performed with two-way ANOVA, followed by Tukey post-hoc testing (*p* < 0.05) to analyze the effect of strength training (ST) and raloxifene (Ral) treatment, and any interactions (ST*Ral). Abbreviations and symbols: +main effect of Ral, #main effect of ST, *interaction of ST plus Ral^a^, vs NT-Veh; ^b^vs NT-Ral; ^c^vs ST-Veh. NT-Ral = non-trained and treated with raloxifene; ST-Veh = strength training and treated with vehicle; ST-Ral = strength training and treated with raloxifene; Ct.Ar = cortical bone area; Ct.Th = average cortical thickness; *I*max = maximum moment of inertia; *I*min = minimum moment of inertia; *J* = polar moment of inertia.

ST, Ral treatment or ST*Ral for 120 days in femoral neck cortical microarchitecture (Fig. 4) of aging female Wistar rats showed that cortical bone area (Ct.Ar) and average cortical thickness (Ct.Th) were affected by ST (p = 0.0115 and p = 0.0294, respectively) and by interaction of treatments (F_(1,15)_ = 14.53, p = 0.0017 and F_(1,11)_ = 8.943, p = 0.0123, respectively). No main effects in NT-Ral group were observed. Furthermore, the maximum moment of inertia (*Imax)* and polar moment of inertia (J) were affected in ST-Veh (p = 0.0010 and p = 0.0311, respectively) and there was an interaction between ST*Ral (F_(1,12)_ = 16.95, p = 0.0014 and F_(1,16)_ = 5.683, p = 0.0299), while no main effects for Ral in NT-Ral group were observed. The minimum moment of inertia (*Imin*) was not affected by treatments.

### Femoral neck maximum load and bone mass after ST or Ral treatment

**Figure 5:**
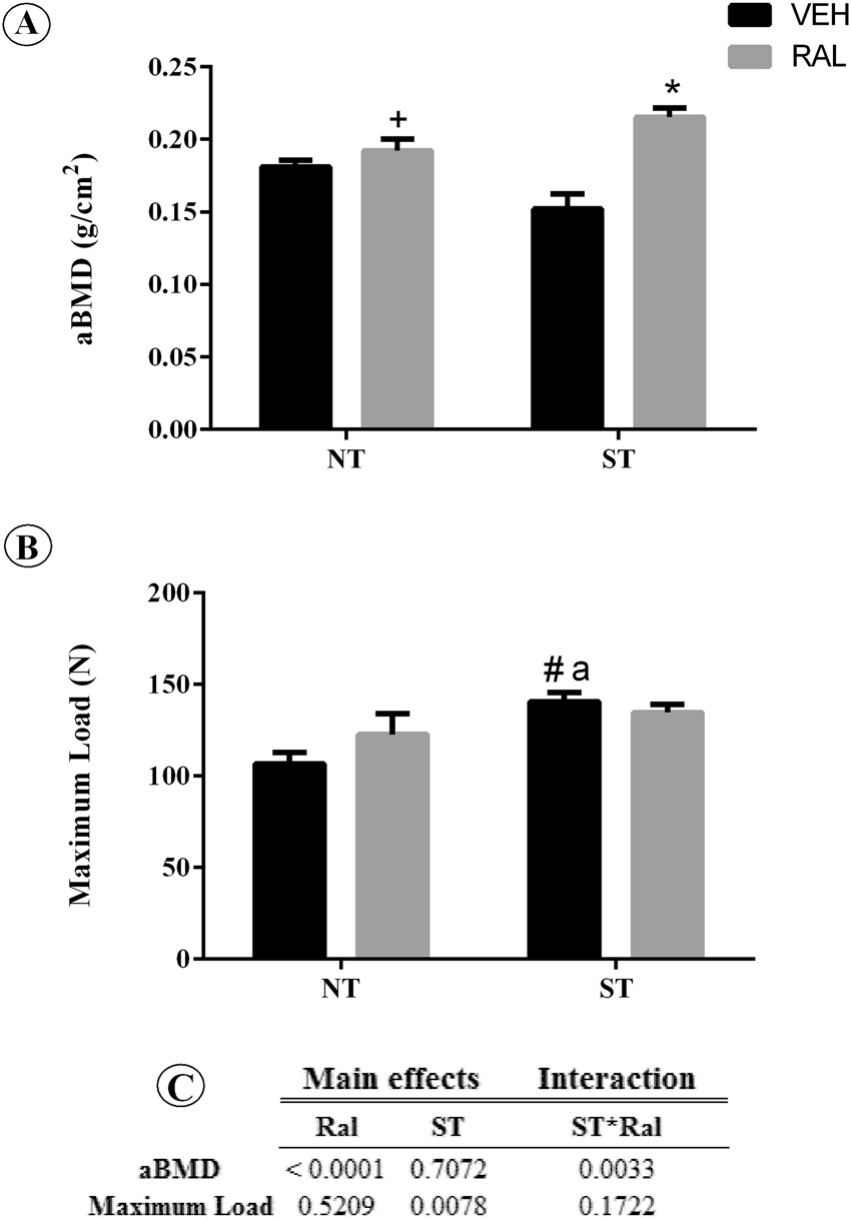
*Ex vivo* areal bone mineral density (aBMD) and maximum load. (**A**) Data of the femoral neck assessed by DXA. (**B**) *Ex vivo* maximum load data of the femoral neck, data assessed by biomechanical compression bending testing. (**C**) Summary of p values in two way ANOVA analysis, n = 7 animals/group. Each column represents mean ± standard error of the mean (SEM). Statistical analysis was performed with two-way ANOVA, followed by Tukey post-hoc testing (*p* < 0.05) to analyze the effect of strength training (ST) and raloxifene (Ral) treatment, and any interactions (ST*Ral). Abbreviations and symbols: + main effect of Ral, # main effect of ST, *interaction of ST plus Ral^a^, vs NT-Veh; ^b^vs NT-Ral; ^c^vs ST-Veh. NT-Veh = non-trained and treated with vehicle; NT-Ral = non-trained and treated with raloxifene; ST-Veh = strength training and treated with vehicle; ST-Ral = strength training and treated with raloxifene.

To evaluate whether ST, Ral or ST*Ral could influence the bone strength, we assessed areal bone mineral density (aBMD) by dual-energy X-ray absorptiometry (DEXA) and maximum load testing measured in femurs (Fig. 5). The Ral treatment (p < 0.0001) and ST*Ral interaction (F_(1,26)_ = 10.46, p = 0.0033) affected the bone mass in femoral neck of aging female Wistar rats. However, maximum load was affected only by ST (p = 0.0078), no main effects of Ral (NT-Ral) or interaction (ST*Ral) between groups were observed. The *pos hoc* test showed that ST-Veh (p = 0.0067) group increased maximum load in comparison to the NT-Veh group.

### Femoral neck bone biomarkers after ST or Ral treatment

**Figure 6:**
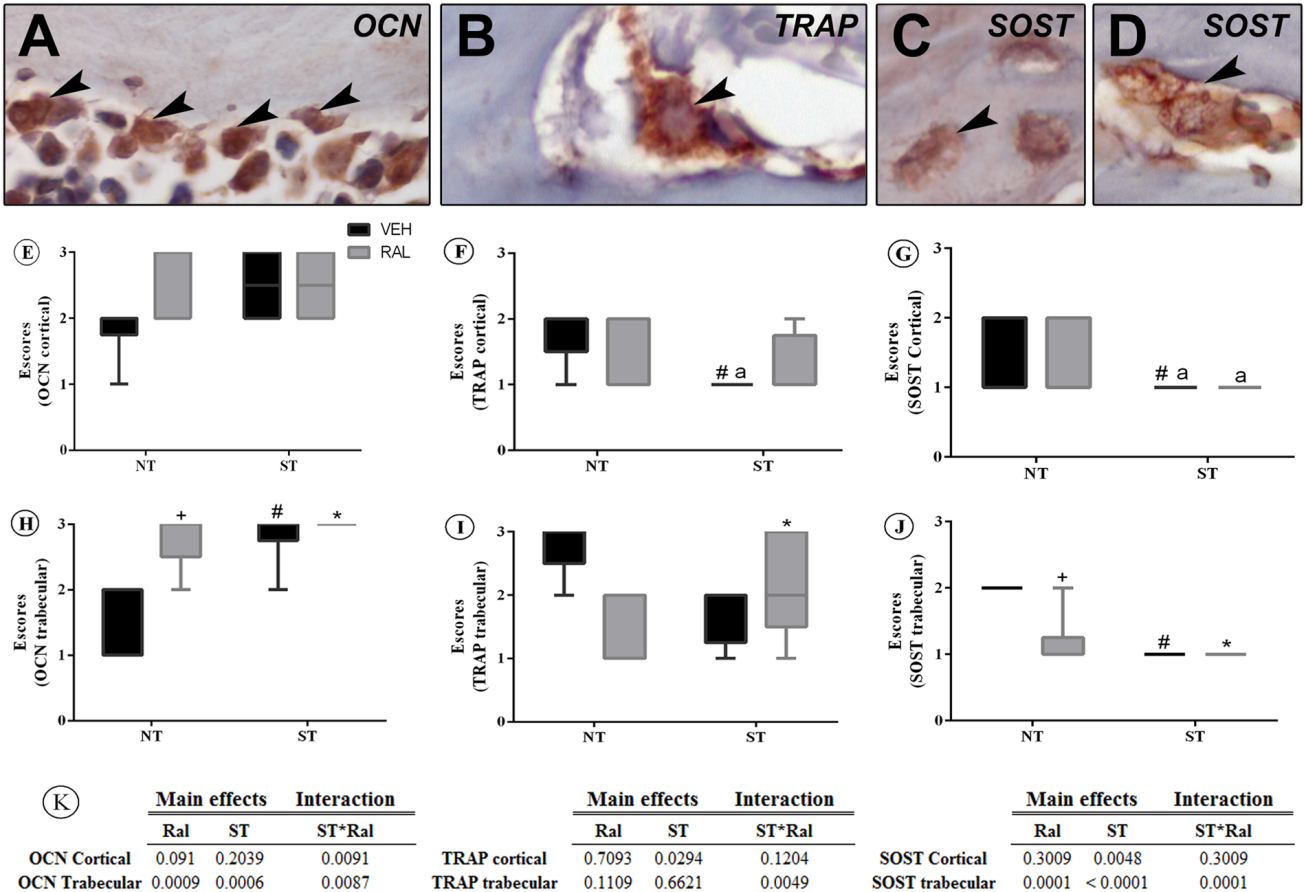
Immunolabeling for OCN, TRAP and SOST assessed by immunohistochemistry. (**A**–**D**) Photomicrographs showing osteoblasts OCN-positive (**A**), osteoclast TRAP-positive (**B**), osteocytes SOST-positive (**C**) and osteoclast SOST-positive. (**E**–**J**): Graphs showing the distribution of scores for OCN (**E** and **H**), TRAP (**F** and **I**) and SOST (**G** and **J**) in cortical and trabecular bone in the different experimental groups. (**K**) summary of p values in two way ANOVA analysis, n = 7 animals/group. Statistical analysis was performed with two-way ANOVA, followed by Tukey post-hoc testing (*p* < 0.05) to analyze the effect of strength training (ST) and raloxifene (Ral) treatment, and any interactions (ST*Ral). Abbreviations and symbols: +main effect of Ral, #main effect of ST, *interaction of ST plus Ral^a^, vs NT-Veh; ^b^vs NT-Ral; ^c^vs ST-Veh. OCN = osteocalcin, TRAP = tartrate-resistant acid phosphatase and SOST = sclerostin. NT-Veh = non-trained and treated with vehicle; NT-Ral = non-trained and treated with raloxifene; ST-Veh = strength training and treated with vehicle; ST-Ral = strength training and treated with raloxifene.

The antibodies used to detect OCN, TRAP and SOST in the immunohistochemical method showed high specificity for these proteins, which was confirmed by the complete absence of immunolabeling in the negative control. Immunolabeling for OCN was predominantly found in the cytosol of osteoblasts, TRAP in multinucleated osteoclasts and SOST in osteocytes and some osteoclasts. The pattern of immunolabeling and immunohistochemical analyses for all biomarkers are shown in Fig. 6.

TRAP (ST-Veh; p = 0.0294) and SOST (p = 0.0048) immunolabeling were decreased in the cortical site of the femoral neck, in the ST group, while no differences were observed after Ral and ST*Ral. However, *pos hoc* analysis showed decreased immunolabeling for cortical TRAP in ST-Veh group (p = 0.0437) compared to NT-Veh group and decreased cortical SOST immunolabeling in ST-Veh (p = 0.0386) and ST-Ral groups (p = 0.0287) in relation to NT-Veh group. Immunolabeling for OCN in this region was not affect by treatments.

In trabecular bone of femoral neck, the interaction of ST and Ral was observed in the immunolabeling for OCN (F_(1,19)_ = 8.549, p = 0.0087), TRAP (F_(1,15)_ = 10.88, p = 0.0049) and SOST (F_(1,19)_ = 22.62, p = 0.0001). Moreover, two way ANOVA showed that Ral and ST affected the immunolabeling for OCN (p = 0.0009; p = 0.0006) and SOST (p = 0.0001; p < 0.0001).

### Femoral neck surface of osteoclasts and osteoblasts

**Figure 7:**
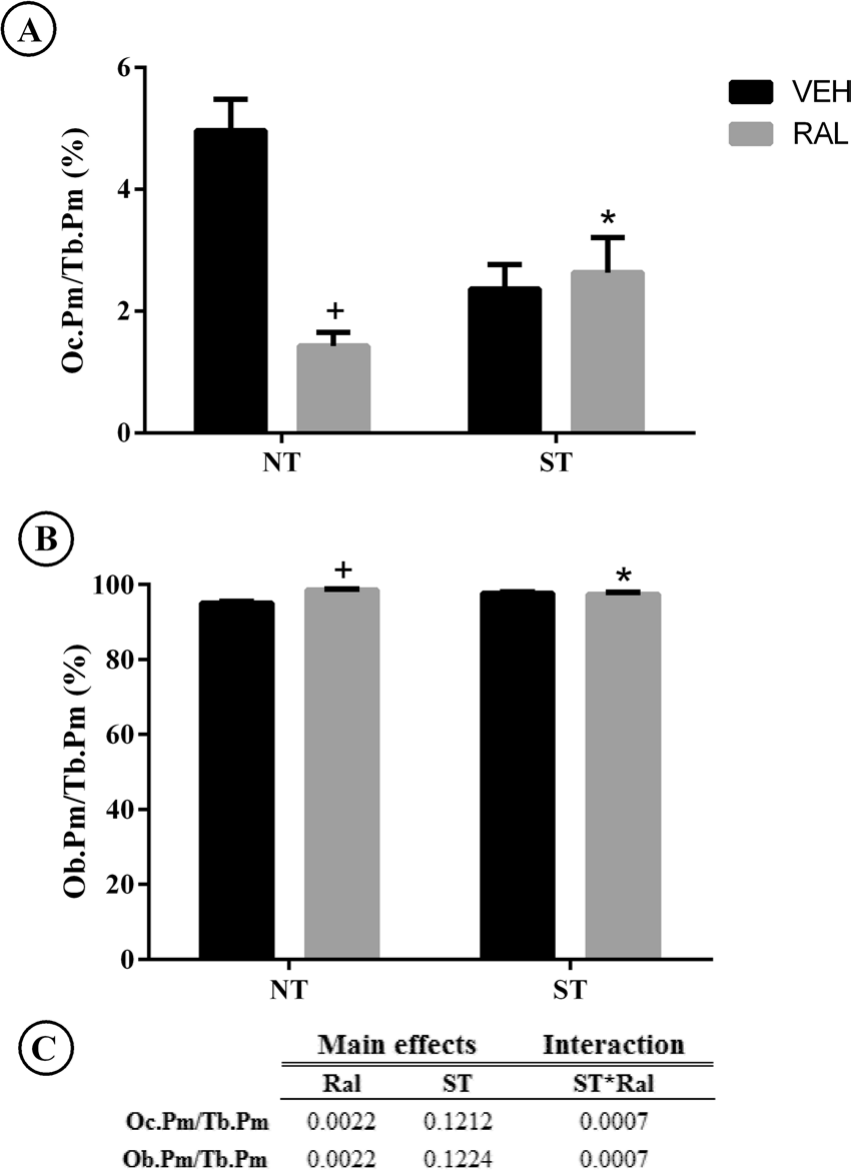
Osteoclast perimeter/Trabecular perimeter (Oc.Pm/Tb.Pm) and Osteoblast perimeter/Trabecular perimeter (Ob.Pm/Tb.Pm) assessed in TRAP positive cells by immunohistochemistry counterstaining with hematoxilin and eosin, analyzed by Image J. (**A**) Oc.Pm/Tb.Pm (%), (**B**) Ob.Pm/Tb.Pm (%), (**C**) summary of p values in two way ANOVA analysis, n = 7 animals/group. Abbreviations and symbols +main effect of Ral, *interaction of ST plus Ral. NT-Veh = non-trained and treated with vehicle; NT-Ral = non-trained and treated with raloxifene; ST-Veh = strength training and treated with vehicle; ST-Ral = strength training and treated with raloxifene.

The analysis of osteoclast (Oc.Pm/Tb.Pm) and osteoblast (Ob.Pm/Tb.Pm) perimeter in trabecular femoral neck (Fig. 7) showed main effects after Ral (NT-Ral; p = 0.0022 and p = 0.0022, respectively) treatment and interaction effects of ST*Ral (F_(1,12)_ = 20.55, p = 0.0007 and F_(1,12)_ = 20.57, p = 0.0007, respectively). Oc.Pm/Tb.Pm and Ob.Pm/Tb.Pm were not significantly affected by ST.

## Discussion

Our results clearly demonstrate that ST and Ral modulate bone remodeling cycle in a different manner, with improved microarchitecture, and are valid options to prevent age-related bone damage. During late periestropause (age 21 months), we observed bone deterioration in the femoral neck of females rats with decreased bone tissue and more separated trabeculae, similar to that found in women during menopause^12^. Concomitantly, decreased cortical polar moment of inertia, predictor of ultimate load^23^, was observed and, together with the trabecular decline, we confirmed the deterioration of the femoral neck in aging rats (see supplementary material).

The results of the present study provide relevant information to the literature, since they show that bone microarchitecture can be a better tool to predict bone strength than bone densitometry. Although BMD assessed by DXA is a widely used tool to prevent fracture risk, it does not directly assess other elements that may contribute to bone strength, such as size, shape, geometry, and amount of bone in the cortical and trabecular compartments. Therefore, the increased aBMD in animals of the NT-Veh and NT-Ral groups could be due to the retention of “old” bone, with loss of heterogeneity, increased size of crystals, what reflects an increased aBMD but not an increased bone strength. Osteoporosis results from decreased bone formation with consequent decrease in acid phosphate replacement, which varies inversely with crystallinity (crystal size and perfection), and this loss of heterogenicity of the material is associated with increased brittleness and increased risk of fracture^24^. Due to the fact that most of the fractures occur in sites with normal BMD, there is the need to consider that other factors may determine bone strength and, thus, fracture risk, other than BMD. In this perspective, QCT allows the 3D reconstruction of the hip and offers a promising alternative for humans to assess aspects of structure more precisely and to evaluate separately the contribution of cortical and trabecular bone to hip fracture risk^25^.

We highlight that the use of maximum voluntary carrying capacity (MVCC) individualizes ST, but measuring the exact strain stimulus that each bone has undergone is a great difficulty, and it is a limitation of studies *in vivo*. However, the capacity of the bone to respond to exercise by improving bone turnover and microarchitecture appears to have a key importance. The adaptive capacity of skeletal tissue provides clear evidence of the impact of mechanical loading, a regulatory process that is further emphasized by the consequences of removing physical signals as reflected by the rapid onset of osteopenia^13,26^. The results of this study showed that mechanical loading plays an important role in bone homeostasis when estrogen levels are decreased. We evaluated the trabecular and cortical bone in the same region, and found that ST can prevent trabecular and cortical deterioration, culminating in an increased maximum load. Considering that the ultimate load and extrinsic stiffness are associated with average principal moment of inertia and maximum polar moment of inertia, the increase in these parameters in the femoral neck of aging rats after ST is very important. Interestingly, this was the only experimental group that showed increased cortical microarchitecture and maximum load, indicating that this parameter is a truly good predictor of ultimate load. The decrease of SOST immunolabeling in the cortical and trabecular femoral neck of aged female rats after ST evidences the modulation of SOST by mechanical loading, and the osteogenic response to physical exercise related to this protein. Through this, we attribute ST as responsible, at least partially, for the fact that ST-Veh animals showed increased bone strength. Therefore, this set of results suggests that bone response to an increased mechanical load favors bone turnover and bone microarchitecture of the femoral neck of rats in the aging period and culminates in better resistance to loading challenges.

Treatment with Ral did not prevent the negative effects verified in aged female, such as minor bone mechanical strength of femoral neck, as much as ST. Ral provided an increase in the trabecular microarchitecture, but it did not affect the cortical parameters or maximum load. Classically, Ral is an antiresorptive drug that diminishes bone turnover. We can confirm this fact with the decrease of plasma TRAP activity and osteoclast surface in this work. This treatment, promoted decreased immunolabeling of SOST in trabecular femoral neck bone of late periestropaused rats, besides increased OCN, as well as decreased perimeter of osteoclasts, corroborating with findings from our lab^14^. Furthermore, cortical bone strength is important for the prevention of fragility fractures since cortical bone represents a substantial amount of the total bone mass in the appendicular skeleton^27^. Thus, after treatment, the analysis of this region showed that Ral had limited effect on cortical bone remodeling in aged rats, since the drug did not decrease SOST in the cortical region of the femoral neck and was not able to prevent the decrease in Ct.Th, resulting in reduced bone and minor structurally effective bone architecture.

Some authors have investigated the role of ST and antiresorptive drugs in bone health and the results did not show an additive effect of the therapies^28,29^. To our knowledge, this study was the first to show that Ral treatment associated with ST provides interactive effects in aging rats bone. This association led to an interactive response in Tb.N, Tb.Sp, aBMD, increased OCN immunolabeling and Ob.Pm/Tb.Pm, decreased TRAP and SOST immunolabeling in the trabecular femoral neck bone, decreased Oc.Pm/Tb.Pm and plasma TRAP activity, showing that the treatments did not act individually for these parameters. Although the interaction between the treatments in the previously mentioned parameters was beneficial, we did not observe improvement in the cortical microarchitecture, perhaps because TRAP immunolabeling did not decrease in this area. All the dynamics of the remodeling process triggered by the association of therapies were not able to respond into improvement of cortical microarchitecture and bone strength. In fact, in this group (ST-Ral), we observed a loss of the response that exercise brought alone. This can be attributed to a strain-related proliferation that is mediated by estrogen receptor ER, in a manner that does not compete with estrogen but can be blocked by ER modulators^30^.

A previous study from our group using rats in initial periestropause (14 to 18 months of age) where estrogen was decreased abruptly by OVX, 120 days of treatment with ST or Ral triggered similar responses in the femoral neck, but the combination of the interventions (ST + Ral) did not bring additional benefits to the bone^14^. In contrast, this study investigated the effects of these treatments in naturally aged animals, during late periestropause (18 to 21 months of age) and found that with a gradual decrease of plasma estrogen, ST can be used to prevent bone loss in this period. Treatment with Ral is also a valid strategy for this prevention, but appears to be less effective than ST, and combining the two therapies provided some additional interactions but did not bring additional effects that justify their dual use.

We can conclude that bone loss in late periestropause can be reversed with the use of ST, as ST alone is able to prevent bone loss and improve cortical bone. The conclusion is that ST can be a systemic intervention for osteoporosis, taking into account the fact that mechanical load generated by ST also affects non-skeletal tissues. However, ST should be used carefully for the prevention and treatment of osteoporosis, because the frequency of exercise and load intensity are essential factors to consider. These results add new information about interventions to prevent age-associated osteoporosis in females and provide good basis for preclinical studies.

## Material and Methods

### Animals

All animal procedures were approved (Protocol Number 2014–00267) by the Institutional Animal Care and Use Committee of the Faculty of Dentistry (Univ. Estadual Paulista – Unesp, Araçatuba, SP, Brazil), and complied with the Guide for Care and Use of Laboratory Animals.

Forty female Wistar rats from the central animal creation of the Faculty of Dentistry of Araçatuba, aged 17–21 month, were housed at 22 °C (±2 °C) and kept under a 12:12 hour light:dark cycle. The animals were allowed free access to water and a commercial pellet diet (Presence® Ratos e Camundongos, Paulínia, SP, Brazil). All female rats were multiparous as this was an inclusion criterion. The reproductive life of these animals was on average from 2 to 8 months of age, reaching 3 to 4 pregnancies. The general health of the rats was monitored on a daily basis and the estrous cycle was checked during the 17^th^ month, to determine acyclicity. Data from our laboratory showed that Wistar female rats in their 18^th^ month had decreased plasma concentrations of estrogen and increased plasma concentrations of luteinizing hormone (LH), characterizing periestropause, which is a period similar to perimenopause in women^31^.

Thus, throughout the 17 months, the experimental animals had their estrous cycles monitored daily, and after confirmation of estrous acyclicity, rats were randomly assigned to one of four groups:1: non-trained plus vehicle (NT-Veh); 2: non-trained plus raloxifene (NT-Ral, n = 10); 3: strength training plus vehicle (ST-Veh); or 4: strength training plus raloxifene (ST-Ral, n = 10). After 120 days from the beginning of the treatments, the animals were sacrificed with an anesthetic overdose and the material was collected for further analysis.

### Raloxifene and vehicle administration

At the beginning of the 18^th^ month, animals in the NT-Ral and ST-Ral groups received 1 mg/kg/day of Ral^14^ (Sigma Aldrich, Munich, Germany) in 0.3 mL of physiological saline solution administered daily by gavage for 120 days. Animals in the other treatment groups received a physiological saline solution (0.3 mL) daily by gavage for the same time period.

### Maximum voluntary carrying capacity (MVCC) and strength training

ST-Veh and ST-Ral groups performed ST with a ladder (1.13 × 0.18 m; 2 cm grid; 80° angle; with a resting area at the top [20 × 20 × 20 cm diameter])^32^, three times per week for 120 days^14^. At the beginning of the 18^th^ month, the animals underwent 1 week of acclimatization. Subsequently, the MVCC of each animal was evaluated with the use of two weighted tubes (steel spheres) attached to the tail. When evaluating MVCC, the initial load was 75% of the body weight of the animal. The animals had two minutes of rest between sets and each new series to be held added 30 grams of weight^32^. This procedure was performed until the animal was not able to fully complete the upward movement. The burden on the previous climb in which the movement failed was considered the maximum stocking rate, and it was used as the basis for the calculation of overhead to be applied during the training period that was initiated forty-eight hours after the MVCC test completion. During 120 days, the animals performed three weekly sessions of ST on alternate days, with each session consisting of six series and two-minute intervals between each series. During the first week, the animals underwent the training with overhead corresponding to 60% of MVCC, which was increased to 70% of MVCC in the second week and 80% of MVCC in the third week. From the 4^th^ week until the end of the proposed period, the animals underwent ST overload at 80% of MVCC. Each month, a new MVCC test was carried out to obtain and maintain the maximum strength capacity of the animals. No animals were excluded due to physical injuries or disorders in the estrous cycle.

### Measurement of body and uterus weight

After 120 days of treatment, all animals were anesthetized (ketamine 80 mg/kg was associated with xylazine 10 mg/kg, intraperitoneally) and weighed. After this, they were sacrificed by decapitation. Immediately after decapitation and collection of femurs, the uteri were weighed on a precision scale to check for possible treatment effects.

### Measurement of plasma levels of estradiol and bone turnover biomarkers

Blood (4 mL) was collected, immediately centrifuged (2256 × g; 15 min; 4 °C), and the plasma was stored (−20 °C). Estradiol levels were measured by employing a rat-specific quantitative sandwich enzyme-linked immunosorbent assay (ELISA, IBL international GMBH, Hamburg, Germany) in accordance with the manufacturer’s instructions. All samples were assayed in duplicate and in the same assay to avoid inter-assay error. The minimum detectable dose of estradiol was 0.28 ng/mL.

The plasma bone formation marker alkaline phosphatase (ALP) was determined using the colorimetric assay that measured the time-dependent formation of p-nitrophenyl (pNP) from pNPP in accordance with a protocol adapted from Chaves Neto *et al*.^33^. The protocol used 25 µL of plasma in a total volume of 0.5 mL containing 2.5 mMpNPP, 2 mM MgCl_2_, 25 mM glycine buffer, pH 9.4. The reaction was initiated by adding the substrate, and it proceeded for 30 min at 37 °C in a water bath. Assay was terminated by adding 0.25 mL of 1 M NaOH solution, and the absorbance determined at 405 nm to determine the amount of pNP formed during the reaction. One unit of enzyme activity is defined as the amount of enzyme that is required to hydrolyze 1 μmol of pNPP per min at 37 °C. Plasma tartrate – resistant acid phosphatase (TRAP) activity was determined by colorimetric assay using protocol adapted from Granjeiro *et al*.^34^ and Janckila *et al*.^35^. Briefly, an aliquot of 25 µL of plasma was assayed in 0.5 mL of reaction mixture consisting of 10 m M*p*-nitrophenyl phosphate (*p*NPP), 100 mM sodium acetate, pH 5.8, 50 mM sodium tartrate and 1 mM*p*-hydroxy mercury benzoate (*p*HMB), the latter acts by inhibiting of low molecular weight acid phosphatases^36^. The reaction was initiated by adding of the substrate, and was usually proceeded for 1 h at 37 °C in a water bath. Assay was terminated by adding 0.25 mL of 1 M NaOH, and the absorbance determined at 405 nm to determine the amount of *p*-nitrophenolate (*p*NP) formed during the reaction. Controls without enzyme were included with each assay to adjust the non-enzymatic hydrolysis of *p*NPP. One unit of enzyme activity is defined as the amount of enzyme that is required to hydrolyze 1 μmol of *p*NPP per min at 37 °C.

### Microtomography

Immediately after being euthanized, the femurs of experimental animals were removed, cleaned with soft tissue, and immediately stored in cryotubes with physiological saline solution at −20 °C for microtomography (micro-CT), bone mineral density and biomechanical compression bending measurements. Twenty-four hours before the micro-CT, the cryotubes with femurs were removed from the −20 °C freezer and placed in the refrigerator (4 °C) to defrost, and 6 hours prior to the micro-CT, they were left at room temperature.

Micro-CT of the femurs was performed using a microtomograph SkyScan 1272 device (SkyScan, Belgium) at the following settings: X-ray voltage 70 kV, X-ray current 143 mA, filter 0.5mm, image pixels size 10 µm, camera resolution setting 2016 × 1344, tomographic rotation 180°, rotation step 0.4°, frame average 3, and scan duration 55 minutes. Each femur was positioned in the cranio-caudal orientation to obtain slices. The images were imported into NRecon software (SkyScan, Leuven, Belgium) and converted from gray scale into Digital Imaging and Communications in Medicine (DICOM) format. To obtain the trabecular and cortical bone of femoral neck, we quantified the volumes of interest (VOI) from the three-dimensional structure of trabecular bone in the femoral neck of all animals and extracted measurements from each dataset in the CT-Analyser (SkyScan, Leuven, Belgium) software. The trabecular and cortical bone of the femoral neck were defined using the polygon selection tool. An interpolation tool was used on the selected region of trabecular and cortical bone. Trabecular bone of the femoral neck was limited by cortical bone in the mediolateral direction. Therefore, greater fidelity for the selected area was acquired using a dynamic interpolation tool. Trabecular and cortical bone was selected and inspected using a binary imaging tool to ensure the use of appropriate threshold values, and was used for all subsequent morphometric analyses. Image processing was required for 3D analysis of the bone morphometric parameters that influence mechanical and structural properties. These parameters were calculated using the CT-Analyser (SkyScan, Leuven, Belgium) 3D software based on a volume model. Segmentation of each femoral neck was performed using the same software.

Abbreviations were used for 3D bone morphometric analysis^37^ and the measurement parameters of micro-CT analyses for trabecular and cortical bone were: bone volume fraction (BV/TV; %), trabecular thickness (Tb.Th; mm), trabecular number (Tb.N; 1/mm), trabecular separation (Tb.Sp; mm), structure model index (SMI), connectivity density (Conn.Dn; 1/mm^3^), cortical bone area (Ct.Ar; mm^2^), average cortical thickness (Ct.Th; mm), maximum moment of inertia (*I*max; mm^4^), minimum moment of inertia (*I*min; mm^4^) and polar moment of inertia (*J*; mm^4^)^38^.

### Bone mineral density measurements

For the areal bone mineral density (aBMD), femurs were thawed and positioned in the frontal plane and anterior posterior view on the scanner table, all oriented the same way, fully scanned in a bowl with 2 cm of water, according to manufacturer instructions. The aBMD of femurs was assessed using dual energy X-ray absorptiometry (Lunar DPX Alpha, WI, USA) and a software for measuring BMD in small animals. The equipment was calibrated according to the manufacturer’s instructions. The same investigator analyzed all scans. For analysis of the femoral neck, a region of interest (ROI) was identified using a square with a known area (0.72 mm^2^), which was located in the femoral neck region of all specimens^14^.

### Biomechanical compression bending testing

The biomechanical properties of the femur were assessed with a compression test, using a Universal Testing Machine (DL 3000, EMIC®, São José dos Pinhais, PR, Brazil). Each femur was placed in a metallic apparatus and maintained in a vertically fixed position (long axis). The load was applied to the area of the femoral head whose vector line of action force was parallel to the long axis of the femur, causing a bending movement in the femoral neck region. The deformation rate was 5 mm/min with standardized parameters of loaded cells set to 2000 N of capacity^39^. Load was applied until the bone fractured. The load and displacement of the machine crossbar was monitored and recorded using device software.

### Immunohistochemistry

Immunohistochemical evaluation of OCN, TRAP and SOST immunolabeling were performed by fixing specimens in 4% formaldehyde for 24 h at room temperature, and decalcifying in 10% ethylenediaminetetraacetic acid (changed weekly) for 8 weeks. Decalcified samples were processed in a conventional manner, embedded in paraffin, and submitted to microtomy (3 µm thick), so that the sections were performed along the coronal plane of the proximal femur.

Histology slides with samples from all experimental groups were then submitted to indirect immunoperoxidase technique and the primary antibodies (Santa Cruz Biotechnology, Santa Cruz, CA, USA) anti-OCN (SC 30044; 1:100), anti-TRAP (SC 30833; 1:200), and anti-SOST (obb 100911; 1:500; Biorbyt, São Francisco, CA, USA). The dilution of primary antibodies was based on a titration test. Immunohistochemical processing followed the protocol described by Stringhetta-Garcia *et al*.^14^.

Histological sections (trabecular and cortical femoral neck bone) were examined under bright field illumination on a light microscope (Optiphot-2, Nikon, Japan) by investigators who were blind to treatment assignments. The scores for TRAP immunolabeling pattern were modified from Stringhetta-Garcia *et al*.^14^: score 3 indicates high pattern, over 8 immunoreactive (IR) cells per area; score 2 indicates moderate pattern, 3 to 7 IR cells per area; score 1 indicates low pattern, less than 3 IR cells per area; and score 0 indicates the absence of immunolabeling. The scores for OCN and SOST immunolabeling patterns were adapted from Stringhetta-Garcia *et al*.^14^: score 3, high pattern, approximately 75% of IR cells per area; score 2, moderate pattern, approximately 50% IR cells per area; score 1, low pattern, approximately 25% IR cells per area; and score 0, absence of immunolabeling. These immunolabeling scores were compared among experimental groups.

### Femoral neck surface of osteoclasts and osteoblasts

The TRAP-positive slides were submitted to the indirect immunoperoxidase technique and were counterstained with hematoxylin and eosin for surface measurement of osteoclasts and osteoblasts. The surface occupied by TRAP-positive osteoclasts, the surface occupied by osteoblast and bone lining cells in the total perimeter of the bone trabeculae were measured using the software *Image J* and were expressed as % of the mean and standard error of the mean.

### Statistical analysis

Statistical analysis was performed using two-way ANOVA. Tukey’s post-hoc test was performed for analyzes where no interactions were observed, *p* values <0.05 were considered statistically significant. Data are presented as mean ± SEM of independent replicates (n = 8–10 animals for physiological parameters, measurement of estradiol, TRAP and ALP; n = 7 for micro-CT, densitometry, biomechanical, immunohistochemistry and osteoblast/osteoclast surface analysis). Statistical analyses were conducted using GraphPad Prism (version 6.01; GraphPad Software, Inc.).

### Institution that approved the experimental protocols

All animal procedures were approved by the Institutional Animal Care and Use Committee of the Faculty of Dentistry of Universidade Estadual Paulista Júlio de Mesquita Filho – UNESP, Araçatuba, SP, Brazil (Protocol Number 2014-00267) and performed in accordance with the Guide for Care and Use of Laboratory Animals.

## Electronic supplementary material

Supplementary Info
