# Supplementary material for: Effects of strength training and raloxifene on femoral neck metabolism and microarchitecture of aging female Wistar rats
Source: Sci Rep. 2017 Oct 31;7:14410. doi: 10.1038/s41598-017-13098-5 (PMC5663961; doi:10.1038/s41598-017-13098-5)
Supplement: Supplementary file 1 — Supplementary Info [file 41598_2017_13098_MOESM1_ESM.pdf]

# Effects of strength training and raloxifene on femoral neck metabolism and microarchitecture of aging female Wistar rats

Camila Tami Stringhetta-Garcia[a,*], Samuel Rodrigues Lourenço Morais[a], Fernanda Fernandes[a], Melise Jacon Perez-Ueno[a], Ricardo de Paula Almeida[a], Mário Jefferson Quirino Louzada[a], Antonio Hernandes Chaves-Neto[a,b], Edilson Ervolino[b], Rita Cássia Menegati Dornelles[a,b]

[a]Programa de Pós-Graduação Multicêntrico em Ciências Fisiológicas; [b]Univ Estadual Paulista (Unesp), Faculdade de Odontologia, Departamento de Ciências Básicas, Araçatuba, 16018-805,Brasil.

*Corresponding author: Department of Basic Sciences, Araçatuba Dental School, UNESP, Rodovia Marechal Rondon, Km 527, CEP 16018-805, Araçatuba, SP, Brazil.

Phone: +55 18 3636-2757.

E-mail address: camilatami@foa.unesp.br (Stringhetta-Garcia, CT)

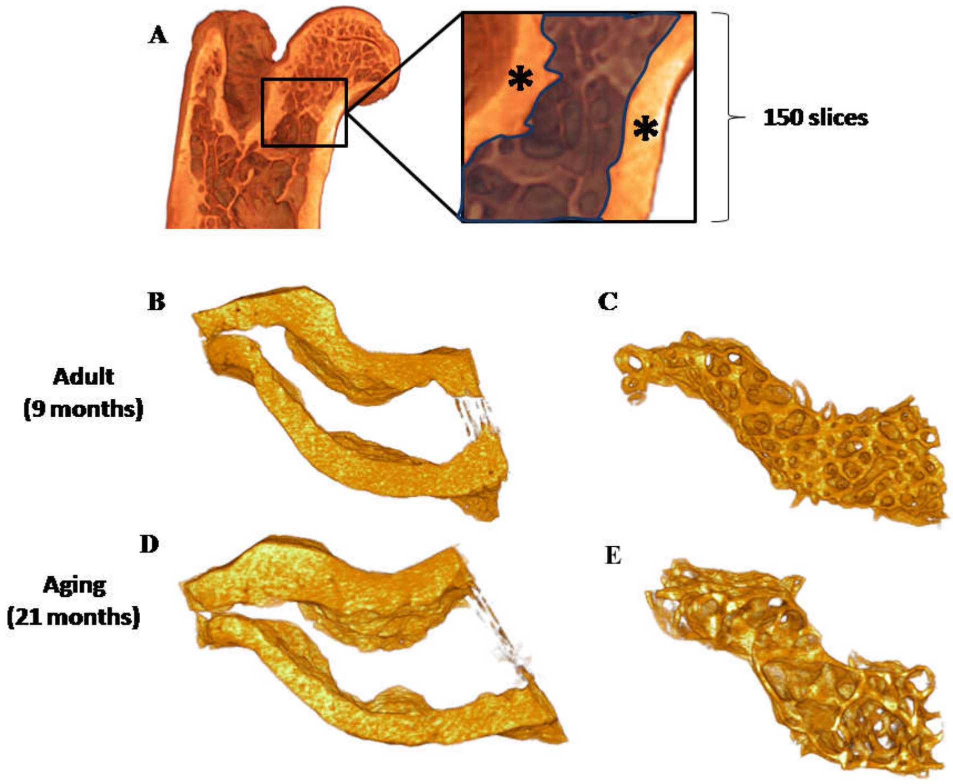


**Figure 1Characterization of 3D morphometric analysis.(A)** Scanned image of proximal epiphysis, with dimensions of the region of interest (femoral neck).The region of interest in trabecular femoral neck is marked by crosshatched blue and cortical femoral neck is marked *. The 3D images show a typical example of trabecular and cortical bone in the femoral neck of adult animals at 9 months of age **(B, C)**, and 21 months of age **(D, E)**.

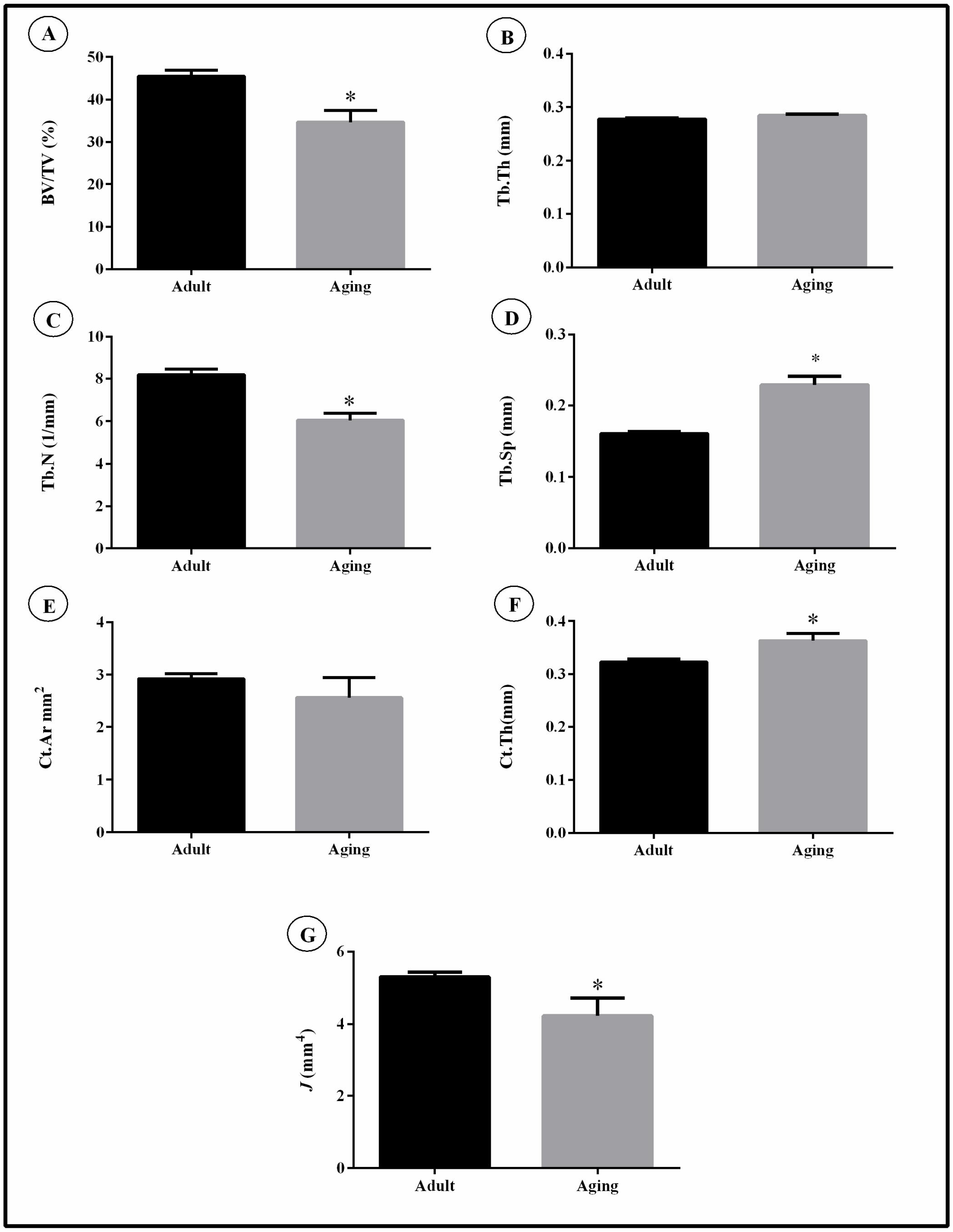


**Figure 2** ***Ex vivo bone microarchitecture***. *Data of the femoral neck of adult 9-month-old and 21-month-old rats, assessed by microCT* ***(A)*** *BV/TV.* ***(B)****Tb.Th.* ***(C)*** *Tb.N.* ***(D)*** *Tb.Sp.* ***(E)*** *Ct.Ar.* **(F)** Ct.Th. **(G)** *J*. Each column represents the mean ± standard error of the mean (SEM). Statistical analysis was performed with Student's test T. Abbreviations and symbols: * *vs Adult;* BV/TV = bone volume fraction; Tb.Th = trabecular thickness; Tb.N = trabecular number; Conn.Dn = connectivity density; Tb.Sp

= trabecular separation; Ct.Ar = cortical bone area; Ct.Th = average cortical thickness; $J$ = polar moment of inertia.
